# Supplementary material for: Dimensional crossover in semiconductor nanostructures
Source: Nat Commun. 2016 Aug 31;7:12726. doi: 10.1038/ncomms12726 (PMC5013694; doi:10.1038/ncomms12726)
Supplement: Supplementary Information — Supplementary Figures 1-6, Supplementary Notes 1-8 and Supplementary References [file ncomms12726-s1.pdf]

Supplementary Figures

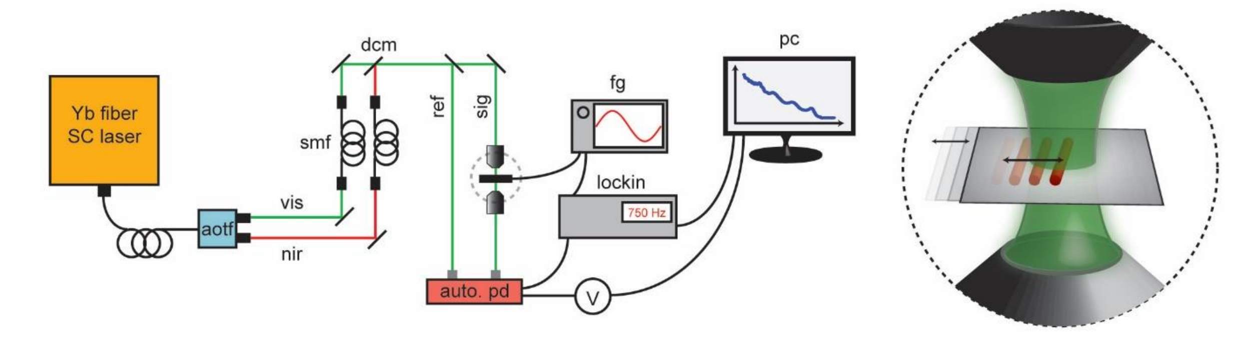

Supplementary Figure 1. Scheme of the experimental design.

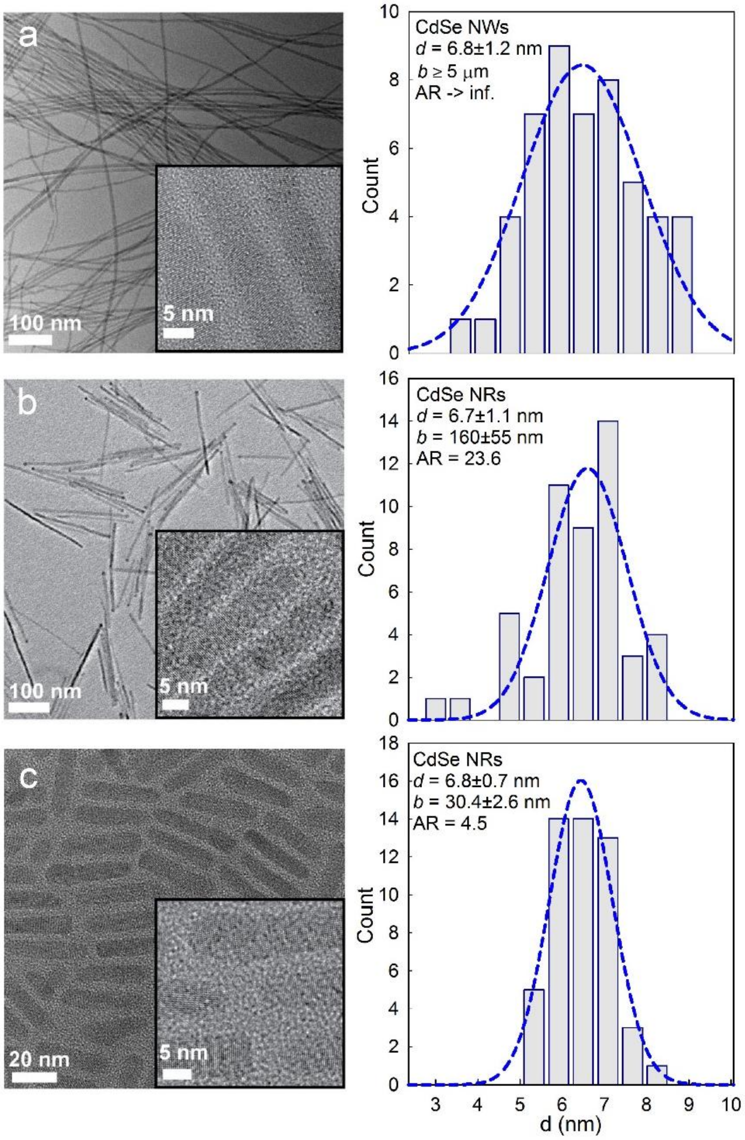

Supplementary Figure 2. Low magnification (high magnification inset) TEM images of CdSe a) NWs, b) b=160±55 nm NRs, and c) b=30.4±2.6 nm NRs. Corresponding diameter distributions

are provided in the rightmost column (grey vertical bars) along with their best Gaussian fits (dashed blue lines).

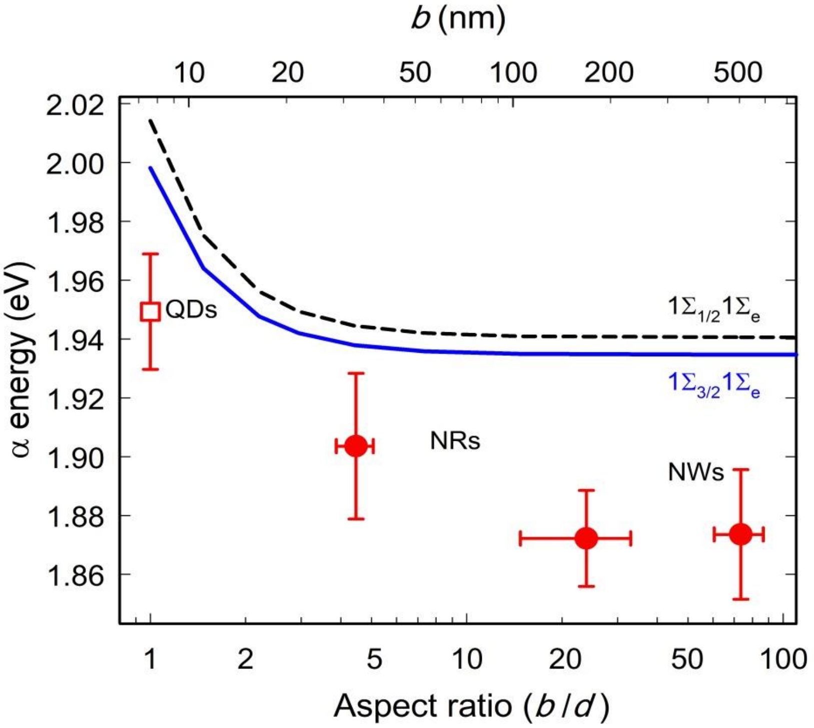

Supplementary Figure 3. QSL energies of the first two (lowest) exciton states ($1\Sigma_{1/2}1\Sigma_e$; black dashed line, and $1\Sigma_{3/2}1\Sigma_e$; blue solid line), as a function of NR aspect ratio (*b*/*d*). For comparison, experimentally determined NW/NR α energies (solid red circles) are also included. Also shown are corresponding literature-derived QD values (open red square).[1,2,3] Standard deviations reported.

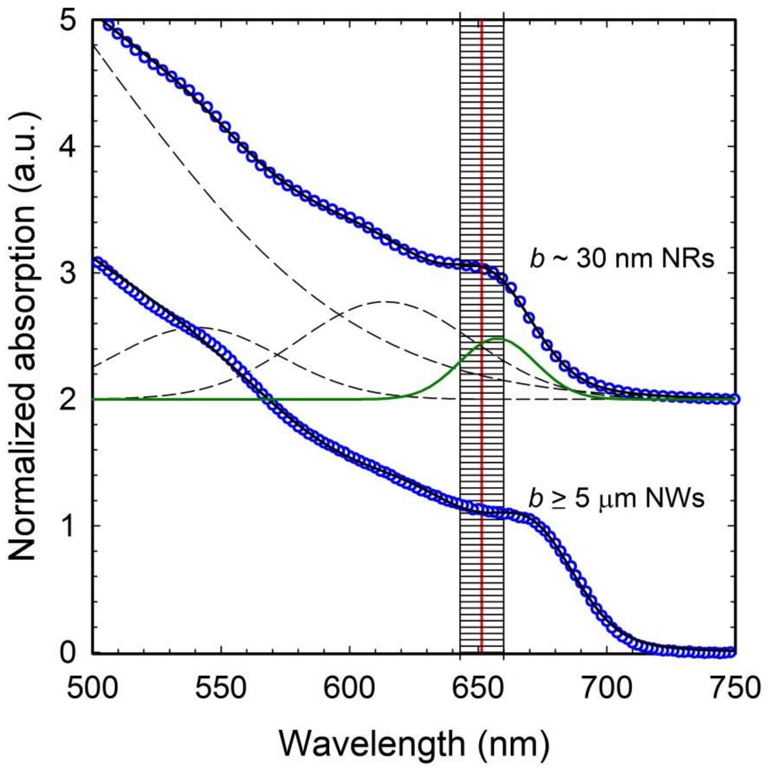

Supplementary Figure 4. Open blue circles represent the ensemble absorption spectrum of *b*~30 nm NRs (top) and *b*≥5 μm NWs (bottom). Data offset for clarity. The NR spectrum is fit to a sum of Gaussians represented by the solid black line, with individual Gaussians shown as solid green (band edge) and dashed grey (higher energy transitions) lines. The vertical red line represents the average single NR α energy for *b*~30 nm NRs while horizontal black lines indicate one standard deviation about the mean.

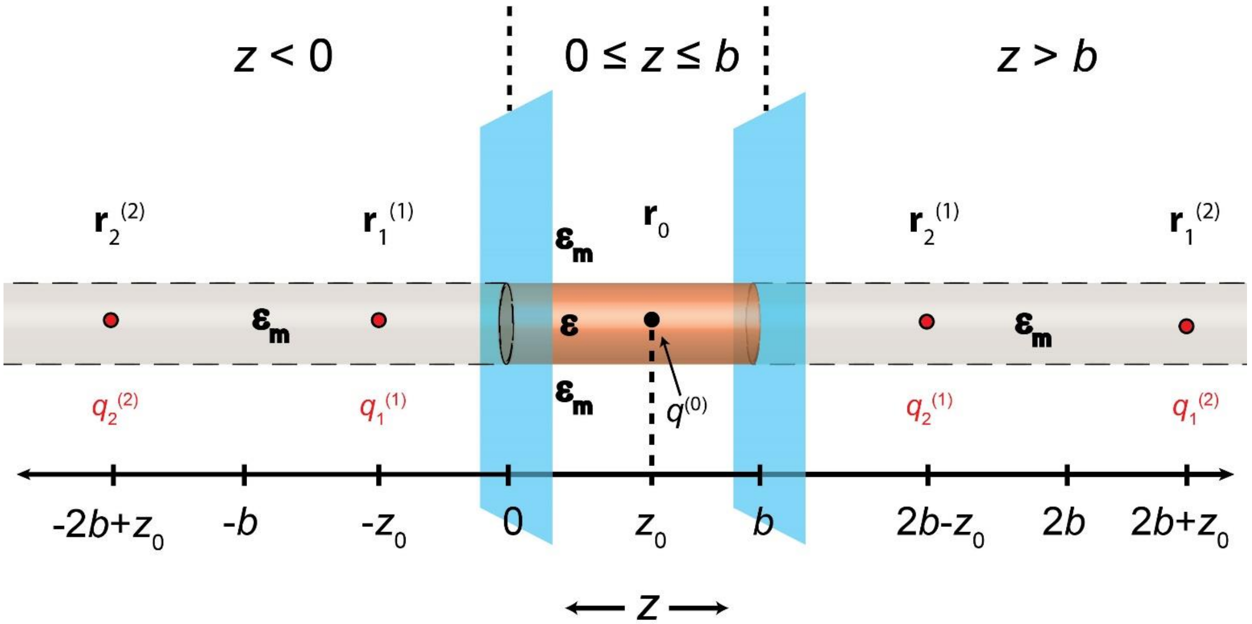

Supplementary Figure 5. Cartoon of finite cylinder scheme used in electrostatic calculations.

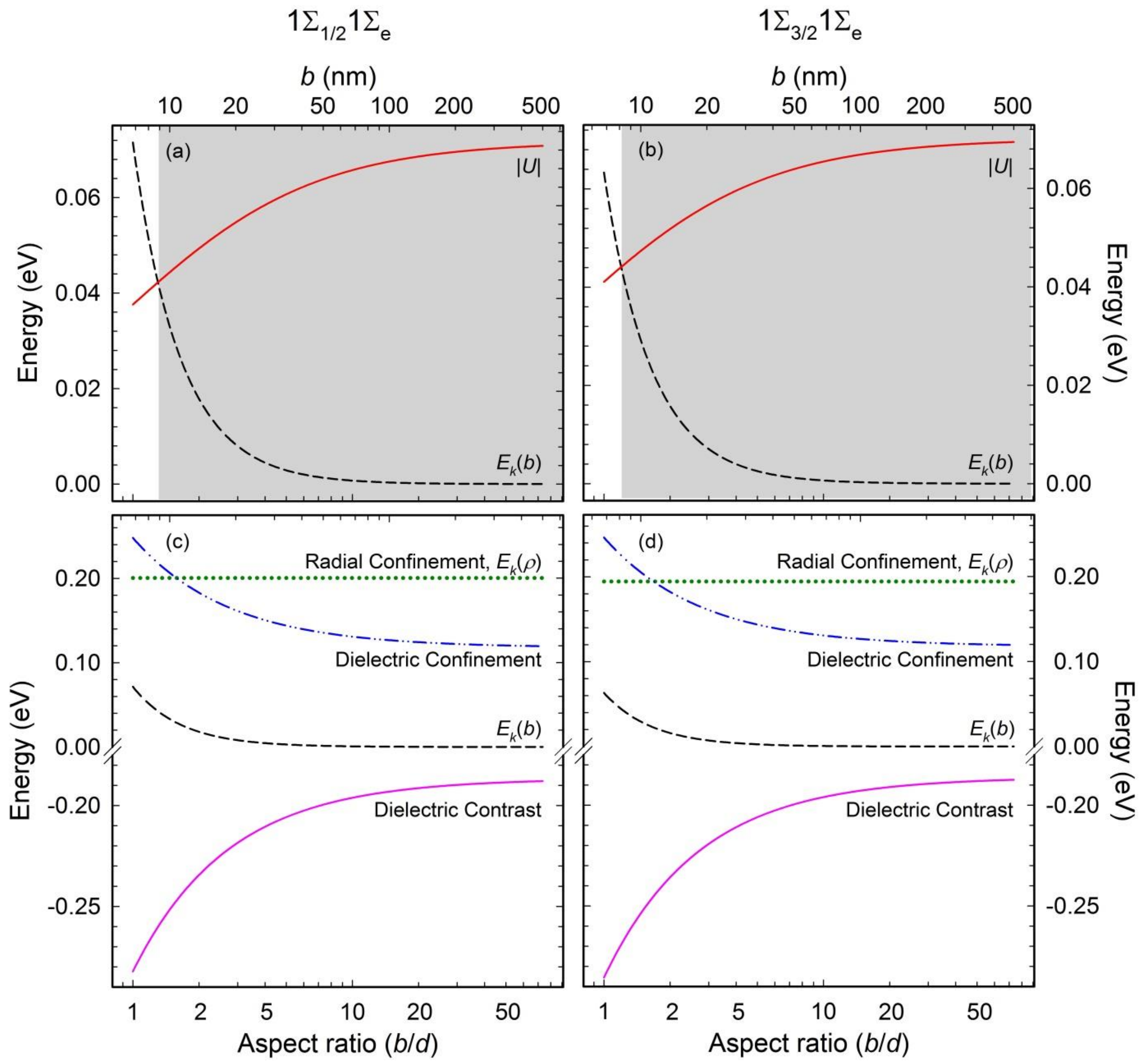

Supplementary Figure 6: (a,b) Longitudinal kinetic energy, $E_k(b)$ (dashed black line) and net electrostatic energy, $|U|$ (solid red line) as functions of aspect ratio for d~6.8 nm NW/NR $1\Sigma_{1/2}1\Sigma_e$ (a) and $1\Sigma_{3/2}1\Sigma_e$ (b) states. White and shaded grey regions represent regions of 0D and 1D confinement respectively. (c,d) Evolution of the various contributions to total transition energies viz. radial [$E_k(\rho)$, dotted green line] and longitudinal [$E_k(b)$, dashed black line] confinement contributions to the total kinetic energy. Also shown are dielectric contrast (solid pink line) and dielectric confinement (dash-dot-dot blue line) contributions to $|U|$ for d~6.8 nm NW/NR $1\Sigma_{1/2}1\Sigma_e$ (c) and $1\Sigma_{3/2}1\Sigma_e$ (d) states.

Supplementary Notes

Supplementary Note 1. Spatial modulation spectroscopy experimental setup

Supplementary Figure 1 shows a schematic of the spatial modulation set-up. A supercontinuum (SC) laser source based on a Yb-doped fiber laser is passed through a dual-crystal acousto-optic tunable filter (AOTF). One crystal provides visible wavelengths (450-700 nm). The other covers the NIR portion of the spectrum (600-1100 nm). These two output channels are passed through single-mode fibers (smf), recombined, and are then split into signal (sig) and reference (ref) paths. The signal beam is directed into an inverted microscope, while the reference beam is focused onto the reference channel of an ultra-low noise autobalanced photodiode (auto. pd). The signal beam is focused on the sample with a high numerical aperture (dry) objective (60x/0.95NA) and is subsequently collected using a second collinear high NA (oil) objective (60x/1.40NA). Finally, the transmitted light is imaged onto the signal channel of the autobalanced photodiode. Spatial modulation is supplied through the amplified sine output of a function generator operating at 750 Hz. The AC coupled output of the autobalanced photodiode is fed into a lockin amplifier (Stanford Research Systems, SRS 830) and the DC portion of the signal is monitored with a digital multimeter (Keithley 2000).

Supplementary Note 2. CdSe nanowire and nanorod synthesis

Nanowire (NW) synthesis

CdSe NWs with d=6.8±1.2 nm were synthesized using a previously established solution-liquid-solid (SLS) growth method.[4] In brief, 25 mg CdO (0.19 mmol), 0.5 g trioctylphosphine oxide (TOPO, 1.3 mmol), and 0.662 g myristic acid (2.9 mmol) were placed into a 3-neck flask. The mixture was held under vacuum at 100 °C for 1 hour to remove any water and oxygen. The vessel was then backfilled with N_2 and the mixture was heated to 310 °C. Upon reaching ~300 °C the

brown-red slurry turned clear, indicating conversion of CdO to cadmium myristate. The temperature of the flask was then decreased to 250 °C. At this point, a mixture of 1 M trioctylphosphine selenide (TOPSe; 25 μL), trioctylphosphine (TOP; 200 μL) and 1 mM $BiCl_3$ in acetone (25 μL) was injected into the flask. The immediate appearance of a brown precipitate indicated the formation of CdSe NWs. The flask was kept at 250 °C for 1 minute at which point the reaction was quenched by rapid cooling. Prior to reaching 100 °C, 5 mL of toluene was added to prevent TOPO from solidifying. The suspension was subsequently allowed to cool to room temperature whereupon a dark brown precipitate was isolated by centrifugation. Several washing steps were carried out which involved rinsing the precipitate in a 70%:30% toluene:methanol solution and recovering the product by centrifugation. The resulting suspension was dispersed in toluene and was stored for future measurements. Transmission electron microscopy (TEM) images reveal samples composed of high-quality, crystalline CdSe NWs with lengths exceeding 10 μm. The diameter distribution was found to be 6.8±1.2 nm (N=50 nanowires). TEM images can be found in Supplementary Figure 2a.

b=160 nm nanorod (NR) synthesis

CdSe nanorods with *d*=6.7±1.1 nm and *b*=160±55 nm were synthesized using a modified solution-liquid-solid (SLS) NW growth method.[4] A 3-neck flask containing TOPO (2.5 g, 6.47 mmol), myristic acid (0.329 g, 1.44 mmol) and CdO (25 mg, 0.19 mmol) was connected to a Schlenk line and was heated under vacuum at 110 °C for 1 hour. The reaction mixture was then backfilled with N_2 and was heated to 350 °C until the solution turned clear. The temperature was then lowered to 250 °C whereupon an injection solution consisting of 1 M TOPSe (25 μL, 25 μmol) and 2 mM $BiCl_3$ in acetone (100 μL, $2x10^{-4}$ mmol) was introduced. The reaction was left at this temperature for 10 seconds whereupon the initially clear solution became dark brown,

indicating NR growth. The mixture was then rapidly cooled to 70-60 °C and was diluted with 5 mL of toluene to prevent TOPO from solidifying. CdSe NRs were precipitated from the resulting suspension by adding 3 mL of methanol followed by centrifugation. The supernatant was discarded and recovered NRs were dispersed in 5 mL of pyridine to remove any excess myristic acid. The rods were subsequently washed several times in a toluene/methanol mixture and were ultimately stored in toluene. TEM images reveal samples composed of high-quality, crystalline CdSe NRs with lengths of b=160±55 nm. The diameter distribution was found to be d=6.7±1.1 nm (N=50 nanorods). TEM images can be found in Supplementary Figure 2b.

b=30 nm NR synthesis

CdSe NRs with d=6.8±0.7 nm and b=30.4±2.6 nm were synthesized using a published procedure.[5] A 3-neck flask containing TOPO (1.0 g, 2.9 mmol), tetradecylphosphonic acid (TDPA, 0.557 g, 2 mmol), di-n-octylphosphinic acid (DOPA, 0.1 g, 0.34 mmol) and CdO (0.128 g, 1 mmol) was connected to a Schlenk line and was heated under vacuum at 110 °C for 1 hour. The reaction mixture was then backfilled with N_2 and was heated to 350-360 °C until the solution turned clear. The temperature was then lowered to 280 °C whereupon a series of four injection solutions consisting of 0.41 M TOPSe (0.6 mL for each injection, 0.247 mmol) was introduced. The time interval between injections was 2 minutes. The reaction was left at this temperature for an additional 30 minutes following the fourth injection. The mixture was then cooled to 60-70 °C and was diluted with 5 mL of toluene to prevent TOPO from solidifying. CdSe NRs were precipitated from the resulting suspension by adding 3 mL of methanol followed by centrifugation. The NRs were subsequently washed three times in a toluene/methanol mixture and were ultimately retained as a toluene suspension. TEM images reveal samples composed of high-quality,

crystalline CdSe NRs with lengths of b=30.4±2.6 nm. The diameter distribution was found to be d=6.8±0.7 nm (N=50 nanorods). TEM images can be found in Supplementary Figure 2c.

Supplementary Note 3. Effective mass model

To describe the evolution of a particle's electronic structure as it changes from 1D to 0D, we have employed a modified version of the 4-band effective mass model previously developed by Efros and Lambrecht.[6] Notably, we do not assume that the kinetic energy associated with longitudinal motion is negligible in comparison to transverse motion (i.e. $k_z \neq 0$), and thus solve for the eigenenergies and eigenvectors of a dielectric cylinder of finite length.

Electron wavefunction

The wavefunction of an electron inside a finite length cylinder is given by

$$\Psi_e = u_{\pm 1/2} R(\rho)\Phi(\phi)Z(z) \quad (1)$$

where $u_{\pm 1/2}$ and $R(\rho)\Phi(\phi)Z(z)$ are its Bloch and envelope functions, respectively. The Hamiltonian in cylindrical coordinates is

$$\hat{H} = \frac{-\hbar^2}{2m^*}\left\{\frac{\partial^2}{\partial \rho^2} + \frac{1}{\rho}\frac{\partial}{\partial \rho} + \frac{\partial^2}{\partial z^2} + \frac{1}{\rho^2}\frac{\partial^2}{\partial \phi^2}\right\}. \quad (2)$$

From Equations 1 and 2, it follows that

$$\hat{H}\Psi_e = E\Psi_e = \frac{-\hbar^2}{2m^*}\left\{\frac{\partial^2 \Psi_e}{\partial \rho^2} + \frac{1}{\rho}\frac{\partial \Psi_e}{\partial \rho} + \frac{\partial^2 \Psi_e}{\partial z^2} + \frac{1}{\rho^2}\frac{\partial^2 \Psi_e}{\partial \phi^2}\right\}$$

$$= \frac{-\hbar^2}{2m^*}\left\{Z(z)\Phi(\phi)\frac{d^2\mathrm{R}(\rho)}{d\rho^2} + Z(z)\Phi(\phi)\frac{1}{\rho}\frac{d\mathrm{R}(\rho)}{d\rho} \quad (3)$$

$$+ \mathrm{R}(\rho)\Phi(\phi)\frac{d^2\mathrm{Z}(z)}{dz^2} + \mathrm{R}(\rho)\mathrm{Z}(z)\frac{1}{\rho^2}\frac{d^2\Phi(\phi)}{d\phi^2}\right\}.$$

Dividing both sides by $R(\rho)\Phi(\phi)Z(z)$, results in

$$E = \frac{-\hbar^2}{2m^*}\left\{\frac{1}{R(\rho)}\frac{d^2\mathrm{R}(\rho)}{d\rho^2} + \frac{1}{R(\rho)}\frac{1}{\rho}\frac{d\mathrm{R}(\rho)}{d\rho} + \frac{1}{\mathrm{Z(z)}}\frac{d^2\mathrm{Z(z)}}{dz^2} + \frac{1}{\Phi(\phi)}\frac{1}{\rho^2}\frac{d^2\Phi(\phi)}{d\phi^2}\right\}. \tag{4}$$

For $Z(z)$, the boundary conditions of the problem stipulate that Ψ_e vanishes at z=0 and z=b, where b is the length of the cylinder. The function $Z(z) = \sin\left(\frac{n_z\pi}{b}z\right)$ satisfies this condition, with n_z the longitudinal quantum number (n_z = 1, 2, 3, …). The associated energy is $E_z = \frac{{n_z}^2\pi^2\hbar^2}{2m^*b^2}$. Additionally, setting $\Phi(\phi)$ to $\Phi(\phi) = e^{im\phi}$ satisfies the angular periodicity (where m is an angular quantum number; m = ±1, ±2, ±3, …).[7] The remaining radial portion thus satisfies the Bessel differential equation

$$\frac{d^2\mathrm{R}(\rho)}{d\rho^2} + \frac{1}{\rho}\frac{d\mathrm{R}(\rho)}{d\rho} + \mathrm{R}(\rho)\left(-\frac{m^2}{\rho^2} + k^2\right) = 0. \tag{5}$$

Solutions to Equation 5 are $R(\rho) = c_1\,\mathrm{J}_m(k\rho) + c_2\,Y_m(k\rho)$ where $\mathrm{J}_m(k\rho)$ and $Y_m(k\rho)$ are Bessel functions of the first and second kind, respectively. $Y_m(k\rho)$ diverges as $\rho \to 0$, so $c_2 = 0$ and $R(\rho) = c_1\,\mathrm{J}_m(k\rho)$. In addition, the boundary conditions are such that the wavefunction must vanish at the NR edge [$R(\rho = a) = 0$ where a is the NR radius]. Thus, $R(\rho = a) = c_1\,\mathrm{J}_m(ka) = 0$ which occurs when $ka = \alpha_{n,m}$ with $\alpha_{n,m}$ the nth root of the mth order Bessel function. This gives $R(\rho) = c_1\,\mathrm{J}_m\left(\frac{\alpha_{n,m}}{a}\rho\right)$.

The resulting electron wavefunction is

$$\Psi_e = u_{\pm 1/2}\, c_1\,\mathrm{J}_m\left(\frac{\alpha_{n,m}}{a}\rho\right)\sin\left(\frac{n_z\pi z}{b}\right)e^{im\phi}. \tag{6}$$

Subsequent normalization results in

$$\Psi_{e,\pm 1/2}^{(n,n_z,m)} = \frac{u_{\pm 1/2}}{a\sqrt{\frac{\pi b}{2}}\ \mathrm{J'}_m(\alpha_{n,m})}\ \mathrm{J}_m\left(\frac{\alpha_{n,m}}{a}\rho\right)\sin\left(\frac{n_z\pi}{b}z\right)e^{im\phi} \tag{7}$$

with a corresponding energy given by

$$E = \frac{\hbar^2}{2m^*}\left(\frac{\alpha_{n,m}^2}{a^2} + \frac{{n_z}^2\pi^2}{b^2}\right) \tag{8}$$

where $m^* = \frac{m_0}{\left\{1+2f+\frac{E_P}{3}\left(\frac{2}{E+E_g}+\frac{1}{E+E_g+\Delta_{so}}\right)\right\}}$.[7] In the expression for m^*, m_0 is the electron rest mass, $f = -1.035$ accounts for remote bands, $E_p = 19.0$ eV is the Kane parameter, $E_g = 1.74$ eV is CdSe's bulk band gap at room temperature, and $\Delta_{so} = 0.42$ eV is CdSe's spin-orbit splitting.

Hole wavefunctions

Within the four-band effective mass approximation, only contributions from the heavy-hole (HH) and light-hole (LH) are considered. The Hamiltonian expressed in the valence band Bloch function basis,[8] $\left\{u_{+3/2} = \left|\frac{3}{2},+\frac{3}{2}\right\rangle = \frac{1}{\sqrt{2}}(X+iY)\uparrow, u_{+\frac{1}{2}} = \left|\frac{3}{2},+\frac{1}{2}\right\rangle = \frac{i}{\sqrt{6}}[(X+iY)\downarrow -2Z\uparrow],\ u_{-\frac{1}{2}} = \left|\frac{3}{2},-\frac{1}{2}\right\rangle = \frac{1}{\sqrt{6}}[(X-iY)\uparrow +2Z\downarrow], u_{-3/2} = \left|\frac{3}{2},-\frac{3}{2}\right\rangle = \frac{i}{\sqrt{2}}(X-iY)\downarrow\right\}$ is therefore given by[6,9]

$$\widehat{H} = \begin{pmatrix} P+Q & L & M & 0 \\ L^* & P-Q & 0 & M \\ M^* & 0 & P-Q & -L \\ 0 & M^* & -L^* & P+Q \end{pmatrix} \tag{9}$$

where

$$P = \frac{\gamma_1 p^2}{2m_0}$$

$$L = -\frac{i\sqrt{3}}{m_0}\gamma p_z p_- \tag{10}$$

$$Q = \frac{\gamma}{2m_0}(p_\perp^2 - 2p_z^2)$$

$$M = \frac{\sqrt{3}}{2m_0}\gamma p_-^2 \, .$$

More explicitly, we have

$$\hat{H} = \begin{pmatrix} (\gamma_1+\gamma)\frac{\hbar^2k^2}{2m_0} + (\gamma_1-2\gamma)\frac{\hbar^2k_z^2}{2m_0} & -\frac{\sqrt{3}\hbar^2kk_z\gamma}{m_0} & -\frac{\sqrt{3}\hbar^2k^2\gamma}{2m_0} & 0 \\ -\frac{\sqrt{3}\hbar^2kk_z\gamma}{m_0} & (\gamma_1-\gamma)\frac{\hbar^2k^2}{2m_0} + (\gamma_1+2\gamma)\frac{\hbar^2k_z^2}{2m_0} & 0 & -\frac{\sqrt{3}\hbar^2k^2\gamma}{2m_0} \\ -\frac{\sqrt{3}\hbar^2k^2\gamma}{2m_0} & 0 & (\gamma_1-\gamma)\frac{\hbar^2k^2}{2m_0} + (\gamma_1+2\gamma)\frac{\hbar^2k_z^2}{2m_0} & \frac{\sqrt{3}\hbar^2kk_z\gamma}{m_0} \\ 0 & -\frac{\sqrt{3}\hbar^2k^2\gamma}{2m_0} & \frac{\sqrt{3}\hbar^2kk_z\gamma}{m_0} & (\gamma_1+\gamma)\frac{\hbar^2k^2}{2m_0} + (\gamma_1-2\gamma)\frac{\hbar^2k_z^2}{2m_0} \end{pmatrix} \tag{11}$$

where $p_\perp = \hbar k$, $p_- = -i\hbar k$, and $p_z = \hbar k_z$; $\gamma = 0.54 - \frac{E_p}{6\,E_g} + \frac{E_p}{6(E_g+E)}$ and $\gamma_1 = 2.14 - \frac{E_p}{3\,E_g} + \frac{E_p}{3(E_g+E)}$ are energy-dependent Luttinger parameters.[7] Upon expressing $\epsilon = \frac{\hbar^2k^2}{2m_0}$, $\epsilon_z = \frac{\hbar^2k_z^2}{2m_0}$, the Hamiltonian becomes

$$\hat{H} = \begin{pmatrix} (\gamma_1+\gamma)\epsilon + (\gamma_1-2\gamma)\epsilon_z - \frac{\Delta_{cr}}{2} & -\sqrt{3}\,2\sqrt{\epsilon\epsilon_z}\gamma & -\sqrt{3}\epsilon\gamma & 0 \\ -\sqrt{3}\,2\sqrt{\epsilon\epsilon_z}\gamma & (\gamma_1-\gamma)\epsilon + (\gamma_1+2\gamma)\epsilon_z + \frac{\Delta_{cr}}{2} & 0 & -\sqrt{3}\epsilon\gamma \\ -\sqrt{3}\epsilon\gamma & 0 & (\gamma_1-\gamma)\epsilon + (\gamma_1+2\gamma)\epsilon_z + \frac{\Delta_{cr}}{2} & \sqrt{3}\,2\sqrt{\epsilon\epsilon_z}\gamma \\ 0 & -\sqrt{3}\epsilon\gamma & \sqrt{3}\,2\sqrt{\epsilon\epsilon_z}\gamma & (\gamma_1+\gamma)\epsilon + (\gamma_1-2\gamma)\epsilon_z - \frac{\Delta_{cr}}{2} \end{pmatrix} \tag{12}$$

with $\Delta_{cr} = 0.025$ eV denoting the crystal field splitting of the HH and LH bands. At this point, an initial check on the Hamiltonian can be performed by setting $k_z = 0$. This reduces $\hat{H}$ to

$$\hat{H}=\begin{pmatrix}(\gamma_1+\gamma)\epsilon-\frac{\Delta_{cr}}{2} & 0 & -\sqrt{3}\epsilon\gamma & 0\\ 0 & (\gamma_1-\gamma)\epsilon+\frac{\Delta_{cr}}{2} & 0 & -\sqrt{3}\epsilon\gamma\\ -\sqrt{3}\epsilon\gamma & 0 & (\gamma_1-\gamma)\epsilon+\frac{\Delta_{cr}}{2} & 0\\ 0 & -\sqrt{3}\epsilon\gamma & 0 & (\gamma_1+\gamma)\epsilon-\frac{\Delta_{cr}}{2}\end{pmatrix} \quad (13)$$

and is the same expression derived by Efros and Lambrecht for infinitely long nanowires.[6]

Eigenvalues of Equation 12 are found by solving

$$0=\begin{vmatrix} B-\lambda & -\sqrt{3}\,2\sqrt{\epsilon\epsilon_z}\gamma & -\sqrt{3}\epsilon\gamma & 0\\ -\sqrt{3}\,2\sqrt{\epsilon\epsilon_z}\gamma & A-\lambda & 0 & -\sqrt{3}\epsilon\gamma\\ -\sqrt{3}\epsilon\gamma & 0 & A-\lambda & \sqrt{3}\,2\sqrt{\epsilon\epsilon_z}\gamma\\ 0 & -\sqrt{3}\epsilon\gamma & \sqrt{3}\,2\sqrt{\epsilon\epsilon_z}\gamma & B-\lambda\end{vmatrix} \quad (14)$$

where $B=(\gamma_1+\gamma)\epsilon+(\gamma_1-2\gamma)\epsilon_z-\frac{\Delta_{cr}}{2}$ and $A=(\gamma_1-\gamma)\epsilon+(\gamma_1+2\gamma)\epsilon_z+\frac{\Delta_{cr}}{2}$. This yields two doubly degenerate eigenvalues

$$\begin{aligned}\lambda_{LH}&=\gamma_1(\epsilon+\epsilon_z)+\sqrt{\left(\frac{\Delta_{cr}}{2}\right)^2-\gamma\Delta_{cr}(\epsilon-2\epsilon_z)+4\gamma^2(\epsilon+\epsilon_z)^2}\\ \lambda_{HH}&=\gamma_1(\epsilon+\epsilon_z)-\sqrt{\left(\frac{\Delta_{cr}}{2}\right)^2-\gamma\Delta_{cr}(\epsilon-2\epsilon_z)+4\gamma^2(\epsilon+\epsilon_z)^2}\end{aligned} \quad (15)$$

with corresponding eigenvectors of

$$HH_1=c_1^{HH}\begin{pmatrix}\frac{2\epsilon_z}{\sqrt{\epsilon\epsilon_z}}\\ \frac{-\left(\frac{\Delta_{cr}}{2}\right)+\gamma(\epsilon-2\epsilon_z)+\xi}{\sqrt{3}\gamma\epsilon}\\ 0\\ 1\end{pmatrix}\begin{matrix}u_{+3/2}\\ u_{+1/2}\\ u_{-1/2}\\ u_{-3/2}\end{matrix} \quad (16)$$

$$HH_2 = c_2^{HH}\begin{pmatrix} \frac{\left(\frac{\Delta_{cr}}{2}\right) - \gamma(\epsilon - 2\epsilon_z) + \xi}{\sqrt{3}\gamma\epsilon} \\ \frac{2\epsilon_z}{\sqrt{\epsilon\epsilon_z}} \\ 1 \\ 0 \end{pmatrix}\begin{matrix} u_{+3/2} \\ u_{+1/2} \\ u_{-1/2} \\ u_{-3/2} \end{matrix} \tag{17}$$

$$LH_1 = c_1^{LH}\begin{pmatrix} \frac{2\epsilon_z}{\sqrt{\epsilon\epsilon_z}} \\ \frac{-\left(\frac{\Delta_{cr}}{2}\right) + \gamma(\epsilon - 2\epsilon_z) - \xi}{\sqrt{3}\gamma\epsilon} \\ 0 \\ 1 \end{pmatrix}\begin{matrix} u_{+3/2} \\ u_{+1/2} \\ u_{-1/2} \\ u_{-3/2} \end{matrix} \tag{18}$$

$$LH_2 = c_2^{LH}\begin{pmatrix} \frac{\left(\frac{\Delta_{cr}}{2}\right) - \gamma(\epsilon - 2\epsilon_z) - \xi}{\sqrt{3}\gamma\epsilon} \\ \frac{2\epsilon_z}{\sqrt{\epsilon\epsilon_z}} \\ 1 \\ 0 \end{pmatrix}\begin{matrix} u_{+3/2} \\ u_{+1/2} \\ u_{-1/2} \\ u_{-3/2} \end{matrix}. \tag{19}$$

In the latter eigenvectors, $\xi = \sqrt{\left(\frac{\Delta_{cr}}{2}\right)^2 - \gamma\Delta_{cr}(\epsilon - 2\epsilon_z) + 4\gamma^2(\epsilon + \epsilon_z)^2}$. Accompanying normalization coefficients c_1^{HH}, c_2^{HH}, c_1^{LH}, c_2^{LH} are

$$c_1^{HH} = \frac{1}{\sqrt{\left(\frac{-\left(\frac{\Delta_{cr}}{2}\right) + \gamma(\epsilon - 2\epsilon_z) + \xi}{\sqrt{3}\gamma\epsilon}\right)^2 + \left(\frac{2\epsilon_z}{\sqrt{\epsilon\epsilon_z}}\right)^2 + 1}}$$

$$c_2^{HH} = \frac{1}{\sqrt{\left(\frac{\left(\frac{\Delta_{cr}}{2}\right) - \gamma(\epsilon - 2\epsilon_z) + \xi}{\sqrt{3}\gamma\epsilon}\right)^2 + \left(\frac{2\epsilon_z}{\sqrt{\epsilon\epsilon_z}}\right)^2 + 1}} \tag{20}$$

$$c_1^{LH} = \frac{1}{\sqrt{\left(\frac{-\left(\frac{\Delta_{cr}}{2}\right)+\gamma(\epsilon-2\epsilon_z)-\xi}{\sqrt{3}\gamma\epsilon}\right)^2+\left(\frac{2\epsilon_z}{\sqrt{\epsilon\epsilon_z}}\right)^2+1}}$$

$$c_2^{LH} = \frac{1}{\sqrt{\left(\frac{\left(\frac{\Delta_{cr}}{2}\right)-\gamma(\epsilon-2\epsilon_z)-\xi}{\sqrt{3}\gamma\epsilon}\right)^2+\left(\frac{2\epsilon_z}{\sqrt{\epsilon\epsilon_z}}\right)^2+1}}.$$

Corresponding hole states are therefore

$$|HH\rangle_1 = c_1^{HH}\begin{pmatrix}\left(2\epsilon_z/\sqrt{\epsilon\epsilon_z}\right)\mathrm{J}_{Fz-3/2}(k_{lh}\rho)e^{i(F_z-3/2)\phi}\sin(k_z z)\\ \alpha\mathrm{J}_{Fz-1/2}(k_{lh}\rho)e^{i(F_z-1/2)\phi}\sin(k_z z)\\ 0\\ \mathrm{J}_{Fz+3/2}(k_{lh}\rho)e^{i(F_z+3/2)\phi}\sin(k_z z)\end{pmatrix}\begin{matrix}u_{+3/2}\\u_{+1/2}\\u_{-1/2}\\u_{-3/2}\end{matrix}$$

$$|HH\rangle_2 = c_2^{HH}\begin{pmatrix}\beta\mathrm{J}_{Fz-3/2}(k_{lh}\rho)e^{i(F_z-3/2)\phi}\sin(k_z z)\\ \left(2\epsilon_z/\sqrt{\epsilon\epsilon_z}\right)\mathrm{J}_{Fz-1/2}(k_{lh}\rho)e^{i(F_z-1/2)\phi}\sin(k_z z)\\ \mathrm{J}_{Fz+1/2}(k_{lh}\rho)e^{i(F_z+1/2)\phi}\sin(k_z z)\\ 0\end{pmatrix}\begin{matrix}u_{+3/2}\\u_{+1/2}\\u_{-1/2}\\u_{-3/2}\end{matrix} \quad (21)$$

$$|LH\rangle_1 = c_1^{LH}\begin{pmatrix}\left(2\epsilon_z/\sqrt{\epsilon\epsilon_z}\right)\mathrm{J}_{Fz-3/2}(k_{hh}\rho)e^{i(F_z-3/2)\phi}\sin(k_z z)\\ \delta\mathrm{J}_{Fz-1/2}(k_{hh}\rho)e^{i(F_z-1/2)\phi}\sin(k_z z)\\ 0\\ \mathrm{J}_{Fz+3/2}(k_{hh}\rho)e^{i(F_z+3/2)\phi}\sin(k_z z)\end{pmatrix}\begin{matrix}u_{+3/2}\\u_{+1/2}\\u_{-1/2}\\u_{-3/2}\end{matrix}$$

$$|LH\rangle_2 = c_2^{LH}\begin{pmatrix} \nu \mathrm{J}_{Fz-3/2}(k_{hh}\rho)e^{i(F_z-3/2)\phi}\sin(k_z z) \\ \left(2\epsilon_z/\sqrt{\epsilon\epsilon_z}\right)\mathrm{J}_{Fz-1/2}(k_{hh}\rho)e^{i(F_z-1/2)\phi}\sin(k_z z) \\ \mathrm{J}_{Fz+1/2}(k_{hh}\rho)e^{i(F_z+1/2)\phi}\sin(k_z z) \\ 0 \end{pmatrix}\begin{matrix} u_{+3/2} \\ u_{+1/2} \\ u_{-1/2} \\ u_{-3/2} \end{matrix}$$

where $\alpha = \frac{-\left(\frac{\Delta cr}{2}\right)+\gamma(\epsilon-2\epsilon_z)+\xi}{\sqrt{3}\gamma\epsilon}$, $\beta = \frac{\left(\frac{\Delta cr}{2}\right)-\gamma(\epsilon-2\epsilon_z)+\xi}{\sqrt{3}\gamma\epsilon}$, $\delta = \frac{-\left(\frac{\Delta cr}{2}\right)+\gamma(\epsilon-2\epsilon_z)-\xi}{\sqrt{3}\gamma\epsilon}$, and $\nu = \frac{\left(\frac{\Delta cr}{2}\right)-\gamma(\epsilon-2\epsilon_z)-\xi}{\sqrt{3}\gamma\epsilon}$.[9] In Equation 21, F_z is the angular momentum projection onto the NW/NR long (z) axis and $u_{+3/2} = \left|\frac{3}{2},+\frac{3}{2}\right\rangle$, $u_{+1/2} = \left|\frac{3}{2},+\frac{1}{2}\right\rangle$, $u_{-1/2} = \left|\frac{3}{2},-\frac{1}{2}\right\rangle$, and $u_{-3/2} = \left|\frac{3}{2},-\frac{3}{2}\right\rangle$ are again valance band Bloch functions.[8] Momenta (k_{lh}, k_{hh}, and k_z[10]) are given by

$$k_{lh} = \sqrt{\frac{2m_0}{\hbar^2}\left[\frac{1}{8\gamma^2-2\gamma_1^2}\left[\gamma(\Delta_{cr}-8\gamma\epsilon_z)+2\gamma_1(-E+\gamma_1\epsilon_z)+\chi\right]\right]}$$

$$k_{hh} = \sqrt{\frac{2m_0}{\hbar^2}\left[\frac{1}{8\gamma^2-2\gamma_1^2}\left[\gamma(\Delta_{cr}-8\gamma\epsilon_z)+2\gamma_1(-E+\gamma_1\epsilon_z)-\chi\right]\right]} \qquad (22)$$

$$k_z = \frac{n_z\pi}{b}$$

where $\chi = \sqrt{16E^2\gamma^2-4E\gamma\gamma_1\Delta_{cr}+\Delta_{cr}(-3\gamma^2\Delta_{cr}+\gamma_1^2\Delta_{cr}-48\gamma^3\epsilon_z+12\gamma\gamma_1^2\epsilon_z)}$.

NR hole wavefunctions are subsequently formed using the linear combinations of states

$$\Psi_h^{(F_z,n_z)} = \mathcal{A}|HH\rangle_1 + \mathcal{B}|HH\rangle_2 + \mathcal{C}|LH\rangle_1 + \mathcal{D}|LH\rangle_2 \qquad (23)$$

where the prefactors $\mathcal{A}$, $\mathcal{B}$, $\mathcal{C}$, and $\mathcal{D}$ are determined by solving

$$\left(\begin{matrix} c_1^{HH}\left(2\epsilon_z/\sqrt{\epsilon\epsilon_z}\right)\mathrm{J}_{Fz-3/2}(k_{hh}\rho)e^{i(F_z-3/2)\phi}\sin(k_z z) & c_2^{HH}\beta\mathrm{J}_{Fz-3/2}(k_{hh}\rho)e^{i(F_z-3/2)\phi}\sin(k_z z) \\ c_1^{HH}\alpha\mathrm{J}_{Fz-1/2}(k_{hh}\rho)e^{i(F_z-1/2)\phi}\sin(k_z z) & c_2^{HH}\left(2\epsilon_z/\sqrt{\epsilon\epsilon_z}\right)\mathrm{J}_{Fz-1/2}(k_{hh}\rho)e^{i(F_z-1/2)\phi}\sin(k_z z) \\ 0 & c_2^{HH}\mathrm{J}_{Fz+1/2}(k_{hh}\rho)e^{i(F_z+1/2)\phi}\sin(k_z z) \\ c_1^{HH}\mathrm{J}_{Fz+3/2}(k_{hh}\rho)e^{i(F_z+3/2)\phi}\sin(k_z z) & 0 \end{matrix}\right.$$

$$\begin{pmatrix} c_1^{LH}\left(2\epsilon_z/\sqrt{\epsilon\epsilon_z}\right)\mathrm{J}_{Fz-3/2}(k_{lh}\rho)e^{i(F_z-3/2)\phi}\sin(k_z z) & c_2^{LH}\nu\mathrm{J}_{Fz-3/2}(k_{hh}\rho)e^{i(F_z-3/2)\phi}\sin(k_z z) \\ c_1^{LH}\delta\mathrm{J}_{Fz-1/2}(k_{lh}\rho)e^{i(F_z-1/2)\phi}\sin(k_z z) & c_2^{LH}\left(2\epsilon_z/\sqrt{\epsilon\epsilon_z}\right)\mathrm{J}_{Fz-1/2}(k_{hh}\rho)e^{i(F_z-1/2)\phi}\sin(k_z z) \\ 0 & c_2^{LH}\mathrm{J}_{Fz+1/2}(k_{hh}\rho)e^{i(F_z+1/2)\phi}\sin(k_z z) \\ c_1^{LH}\mathrm{J}_{Fz+3/2}(k_{lh}\rho)e^{i(F_z+3/2)\phi}\sin(k_z z) & 0 \end{pmatrix}\begin{pmatrix}\mathcal{A}\\ \mathcal{B}\\ \mathcal{C}\\ \mathcal{D}\end{pmatrix} = \begin{pmatrix}0\\0\\0\\0\end{pmatrix} \tag{24}$$

and in practice, this is done by setting the determinant of the above system of equations to be zero at $\rho = a$. Solving for $\mathcal{A}$, $\mathcal{B}$, $\mathcal{C}$, and $\mathcal{D}$ then yields

$$\mathcal{A} = -\frac{\mathcal{C}c_1^{LH}\mathrm{J}_{Fz+3/2}(k_{lh}a)}{c_1^{HH}\mathrm{J}_{Fz+3/2}(k_{hh}a)}$$

$$\mathcal{B} = -\frac{\mathcal{D}c_2^{LH}\mathrm{J}_{Fz+1/2}(k_{lh}a)}{c_2^{HH}\mathrm{J}_{Fz+1/2}(k_{hh}a)}$$

$$\mathcal{C} = \mathcal{C} \tag{25}$$

$$\mathcal{D} = \frac{\mathcal{C}c_1^{LH}(2\epsilon_z/\sqrt{\epsilon\epsilon_z})\left[\frac{\mathrm{J}_{Fz-3/2}(k_{hh}a)}{\mathrm{J}_{Fz+3/2}(k_{hh}a)}\mathrm{J}_{Fz+3/2}(k_{lh}a) - \mathrm{J}_{Fz-3/2}(k_{lh}a)\right]}{c_2^{LH}\left[\nu\mathrm{J}_{Fz-3/2}(k_{lh}a) - \beta\frac{\mathrm{J}_{Fz-3/2}(k_{hh}a)}{\mathrm{J}_{Fz+1/2}(k_{hh}a)}\mathrm{J}_{Fz+1/2}(k_{lh}a)\right]}\,.$$

Finally, $\mathcal{C}$ is determined by normalizing the NR wavefunction

$$1 = \int_0^{2\pi} d\phi \int_0^{b} dz \int_0^{a} \rho d\rho\, \Psi_h^{(F_z,n_z)^*}\,\Psi_h^{(F_z,n_z)}. \tag{26}$$

Associated NR quantum size level energies (QSLs) are found as roots of the following determinant

$$\begin{vmatrix} c_1^{HH}\left(2\epsilon_z/\sqrt{\epsilon\epsilon_z}\right)\mathrm{J}_{Fz-3/2}(k_{hh}\rho)e^{i(F_z-3/2)\phi}\sin(k_z z) & c_2^{HH}\beta\mathrm{J}_{Fz-3/2}(k_{hh}\rho)e^{i(F_z-3/2)\phi}\sin(k_z z) \\ c_1^{HH}\alpha\mathrm{J}_{Fz-1/2}(k_{hh}\rho)e^{i(F_z-1/2)\phi}\sin(k_z z) & c_2^{HH}\left(2\epsilon_z/\sqrt{\epsilon\epsilon_z}\right)\mathrm{J}_{Fz-1/2}(k_{hh}\rho)e^{i(F_z-1/2)\phi}\sin(k_z z) \\ 0 & c_2^{HH}\mathrm{J}_{Fz+1/2}(k_{hh}\rho)e^{i(F_z+1/2)\phi}\sin(k_z z) \\ c_1^{HH}\mathrm{J}_{Fz+3/2}(k_{hh}\rho)e^{i(F_z+3/2)\phi}\sin(k_z z) & 0 \end{vmatrix}$$

$$\begin{vmatrix} c_1^{LH}\left(2\epsilon_z/\sqrt{\epsilon\epsilon_z}\right)\mathrm{J}_{Fz-3/2}(k_{lh}\rho)e^{i(F_z-3/2)\phi}\sin(k_z z) & c_2^{LH}\nu\mathrm{J}_{Fz-3/2}(k_{hh}\rho)e^{i(F_z-3/2)\phi}\sin(k_z z) \\ c_1^{LH}\delta\mathrm{J}_{Fz-1/2}(k_{lh}\rho)e^{i(F_z-1/2)\phi}\sin(k_z z) & c_2^{LH}\left(2\epsilon_z/\sqrt{\epsilon\epsilon_z}\right)\mathrm{J}_{Fz-1/2}(k_{hh}\rho)e^{i(F_z-1/2)\phi}\sin(k_z z) \\ 0 & c_2^{LH}\mathrm{J}_{Fz+1/2}(k_{hh}\rho)e^{i(F_z+1/2)\phi}\sin(k_z z) \\ c_1^{LH}\mathrm{J}_{Fz+3/2}(k_{lh}\rho)e^{i(F_z+3/2)\phi}\sin(k_z z) & 0 \end{vmatrix} = 0. \quad (27)$$

Equation 27 is used to determine the QSLs for F_z = 1/2 and F_z = 3/2 as a function of aspect ratio. These energies, in conjunction with electron QSL energies (Equation 8) are used to construct the QSL energy gaps for the first and second excited states of CdSe NWs and NRs. The first state is a transition between the lowest hole state, F_z = 3/2, and the first electron state (1S, n=1, m=0) and is denoted here as $1\Sigma_{3/2}1\Sigma_e$. Correspondingly, the next state is the transition between the F_z = 1/2 hole and the 1S electron and is labeled $1\Sigma_{1/2}\ 1\Sigma_e$.

The QSL energy gaps are plotted as a function of aspect ratio in Supplementary Figure 3. Experimentally determined NW/NR α energies (solid red circles) along with corresponding literature-derived QD values (open red square) are also plotted.[1,2,3] As can be seen from the image, at this point, the theory is in poor agreement with the experimental data. However, this is in keeping with prior studies which show that it is necessary to account for the Coulombic forces that act on excited carriers in CdSe NWs and NRs.

Supplementary Note 4. Electrostatic Contributions

Electrostatic forces experienced by photogenerated carriers within NWs/NRs are now included to more accurately describe the particle's electronic structure as a function of aspect ratio.[7,11] This is accomplished by first solving Poisson's equation inside an infinitely long dielectric cylinder. The obtained solution is then used in conjunction with the method of images to solve for electrostatic interactions inside a finite length NW/NR.

The potential at $\mathbf{r} = (\rho, \phi, z)$ due to a point charge (q) at position $\mathbf{r_0} = (\rho_0, \phi_0, z_0)$ inside an infinitely long dielectric NW is given by[11,12]

$$V(\mathbf{r}, \mathbf{r_0}) = \frac{q}{4\pi\varepsilon\varepsilon_0|\mathbf{r} - \mathbf{r_0}|} + \frac{q}{2\pi^2}\sum_{g=-\infty}^{\infty}\int_0^{\infty} du\ e^{ig(\phi-\phi_0)}\ \cos[u(z - z_0)]\ \gamma_g I_g(u\rho) I_g(u\rho_0) \tag{28}$$

where

$$\gamma_g = \frac{(\varepsilon - \varepsilon_m)K_g'(ua)K_g(ua)}{\varepsilon_m I_g(ua)K_g'(ua) - \varepsilon I_g'(ua)K_g(ua)}. \tag{29}$$

Here, q is the elementary charge, ε_0 is the permittivity of free space, ε is the NW's dielectric constant, ε_m is the surrounding's dielectric constant, and a is the NW radius.[11] $I_g(x)$ and $K_g(x)$ are modified Bessel functions of the first and second kind, respectively. In Equation 28, integer g and integration variable u come about from the Green's function expansion (more information can be found in section 3.10 of Jackson's *Classical Electrodynamics*, Ref. 12). The first term of Equation 28 is the potential of a point charge in a bulk dielectric, while the second term accounts for its interaction with the cylinder's dielectric interface.[11] The electrostatic energy of an electron and hole inside the NW is thus given by[11]

$$U(\mathbf{r_e}, \mathbf{r_h}) = \frac{1}{2}\int_{-\infty}^{\infty} d\mathbf{r}\,[q\delta(\mathbf{r} - \mathbf{r_e}) - q\delta(\mathbf{r} - \mathbf{r_h})][V(\mathbf{r}, \mathbf{r_e}) - V(\mathbf{r}, \mathbf{r_h})]. \tag{30}$$

To account for finite NR lengths, Equation 28 is modified via the method of images. First, the infinite cylinder is segmented into three regions along its length, $z<0$, $0\leq z\leq b$, and $z>b$. These segments are assigned dielectric constants of ε_m ($z<0$), ε ($0\leq z\leq b$), and ε_m ($z>b$). This forms a finite cylinder of length b and of dielectric constant ε surrounded by a dielectric medium ε_m (Supplementary Figure 5).

The potential of a point charge inside the cylinder due to the dielectric boundaries at *z*=0 and *z*=*b* is solved via the method of images. A cartoon representation is given in Supplementary Figure 5. Here, mirror image charges (red dots, $q_i^{(n)}$) are placed at $\mathbf{r}_i^{(n)} = \left(\rho_0, \phi_0, z_i^{(n)}\right)$ relative to the point charge's position (black dot) at $\mathbf{r_0} = (\rho_0, \phi_0, z_0)$ within the NR. Image charges are formed at each boundary plane, giving two images (indexed by *i*) for each subsequent number (indexed by *n*) that get successively further away from the boundary surface. Additionally, the magnitude of each image charge is given by $q_i^{(n)} = \left(-\frac{\varepsilon_m - \varepsilon}{\varepsilon_m + \varepsilon}\right)^n q$ to account for the dielectric difference between the rod and the surrounding medium.

The resulting potential at **r** due to a point charge at $\mathbf{r_0}$ inside a dielectric cylinder of length *b* is thus

$$\boxed{V_{b<\infty}(\mathbf{r}, \mathbf{r_0}) = V(\mathbf{r}, \mathbf{r_0}) + \sum_{n,i} V_i^{(n)}(\mathbf{r}, \mathbf{r}_i^{(n)})} \tag{31}$$

where

$$V_i^{(n)}\left(\mathbf{r}, \mathbf{r}_i^{(n)}\right) = \frac{q_i^{(n)}}{4\pi\varepsilon\varepsilon_0\left|\mathbf{r} - \mathbf{r}_i^{(n)}\right|} + \frac{q_i^{(n)}}{2\pi^2}\sum_{g=-\infty}^{\infty}\int_0^{\infty} du\ e^{ig(\phi-\phi_0)}\ \cos\left[u(z - z_i^{(n)})\right]\gamma_g I_g(u\rho) I_g(u\rho_0) \tag{32}$$

and $V(\mathbf{r}, \mathbf{r_0})$ is from Equation 28. Equation 31 is summed over $n = 2j$ and $n = 2j + 1$ ($j =$ 0,1,2,3, ...) so that both even and odd orders of *n* are accounted for, along with $i = 1,2$. The index *n* sums image charges that are successively further away from the NR/medium interface, while the

index *i* sums over charges formed at opposite ends of the NR. Inserting Equation 31 into Equation 30 gives the electrostatic energy of an electron and hole inside a finite dielectric cylinder,

$$U(\mathbf{r_e},\mathbf{r_h}) = U^{(0)}(\mathbf{r_e},\mathbf{r_h}) + \sum_{n,i} U_i^{(n)}(\mathbf{r_e},\mathbf{r_h}). \tag{33}$$

In Equation 33, $U^{(0)}(\mathbf{r_e},\mathbf{r_h})$ is

$$U^{(0)}(\mathbf{r_e},\mathbf{r_h}) = U_e{}^0(\mathbf{r_e}) + U_h{}^0(\mathbf{r_h}) + U_{dir}{}^0(\mathbf{r_e},\mathbf{r_h}) + U_{indir}{}^0(\mathbf{r_e},\mathbf{r_h}) \tag{34}$$

where[11]

$$\begin{aligned} U_e{}^0(\mathbf{r_e}) &= \frac{q^2}{4\pi^2}\sum_{g=-\infty}^{\infty}\int_0^\infty du\, \gamma_g I_g(u\rho_e)^2 \\ U_h{}^0(\mathbf{r_h}) &= \frac{q^2}{4\pi^2}\sum_{g=-\infty}^{\infty}\int_0^\infty du\, \gamma_g I_g(u\rho_h)^2 \\ U_{dir}^0(\mathbf{r_e},\mathbf{r_h}) &= -\frac{q^2}{4\pi\varepsilon\varepsilon_0|\mathbf{r_e}-\mathbf{r_h}|} \\ U_{indir}^0(\mathbf{r_e},\mathbf{r_h}) &= -\frac{q^2}{2\pi^2}\sum_{g=-\infty}^{\infty}\int_0^\infty du\ \cos[u|z_e - z_h|]\ \gamma_g I_g(u\rho_e) I_g(u\rho_h). \end{aligned} \tag{35}$$

Likewise, $U_i^{(n)}(\mathbf{r_e},\mathbf{r_h})$ in Equation 33 is

$$U_i^{(n)}(\mathbf{r_e},\mathbf{r_h}) = U_{e_i}^{(n)}(\mathbf{r_e}) + U_{h_i}^{(n)}(\mathbf{r_h}) + U_{dir_i}^{(n)}(\mathbf{r_e},\mathbf{r_h}) + U_{indir_i}^{(n)}(\mathbf{r_e},\mathbf{r_h}) \tag{36}$$

where

$$\begin{aligned} U_{e_i}^{(n)}(\mathbf{r_e}) &= \frac{q\, q_i^{(n)}}{8\pi\varepsilon\varepsilon_0\left|\mathbf{r_e} - \mathbf{r}_{\mathbf{e}_i}^{(n)}\right|} + \frac{q\, q_i^{(n)}}{4\pi^2}\sum_{g=-\infty}^{\infty}\int_0^\infty du \cos\left[u(z_e - z_{e_i}^{(n)})\right]\gamma_g I_g(u\rho_e)^2 \\ U_{h_i}^{(n)}(\mathbf{r_h}) &= \frac{q\, q_i^{(n)}}{8\pi\varepsilon\varepsilon_0\left|\mathbf{r_h} - \mathbf{r}_{\mathbf{h}_i}^{(n)}\right|} + \frac{q\, q_i^{(n)}}{4\pi^2}\sum_{g=-\infty}^{\infty}\int_0^\infty du \cos\left[u(z_h - z_{h_i}^{(n)})\right]\gamma_g I_g(u\rho_h)^2 \end{aligned} \tag{37}$$

$$U_{dir}{}_i^{(n)}(\mathbf{r_e},\mathbf{r_h}) = -\frac{qq_i^{(n)}}{8\pi\varepsilon\varepsilon_0\left|\mathbf{r_e}-\mathbf{r_h}{}_i^{(n)}\right|} - \frac{qq_i^{(n)}}{8\pi\varepsilon\varepsilon_0\left|\mathbf{r_h}-\mathbf{r_e}{}_i^{(n)}\right|}$$

$$U_{indir}{}_i^{(n)}(\mathbf{r_e},\mathbf{r_h}) = -\frac{qq_i^{(n)}}{4\pi^2}\sum_{g=-\infty}^{\infty}\int_0^{\infty} du\,\gamma_g\left[e^{ig\left(\phi_e-\phi_{h}{}_i^{(n)}\right)}\cos\left[u\left(z_e - z_{h}{}_i^{(n)}\right)\right] I_g(u\rho_e) I_g\left(u\rho_{h}{}_i^{(n)}\right) + e^{ig\left(\phi_h-\phi_{e}{}_i^{(n)}\right)}\cos\left[u\left(z_h - z_{e}{}_i^{(n)}\right)\right] I_g(u\rho_h) I_g\left(u\rho_{e}{}_i^{(n)}\right)\right].$$

Equation 33 is composed of four terms: (1) the electron self-energy $[U_e{}^0(\mathbf{r_e}) + U_{e}{}_i^{(n)}(\mathbf{r_e})]$, (2) the hole self-energy $[U_h{}^0(\mathbf{r_h}) + U_{h}{}_i^{(n)}(\mathbf{r_h})]$, (3) the direct Coulombic attraction $[U_{dir}^0(\mathbf{r_e},\mathbf{r_h}) + U_{dir}{}_i^{(n)}(\mathbf{r_e},\mathbf{r_h})]$, and (4) the indirect attraction of a carrier with its opposite image charge $[U_{indir}^0(\mathbf{r_e},\mathbf{r_h}) + U_{indir}{}_i^{(n)}(\mathbf{r_e},\mathbf{r_h})]$. Terms (1) and (2) are repulsive forces brought about by the NR/medium dielectric interface. Terms (3) and (4) are attractive forces enhanced by reduced screening in the medium's lower dielectric environment. Dielectric confinement is therefore due to terms (1) and (2), while dielectric contrast is due to terms (3) and (4).

The electron self-energy is averaged over the electron probability density to yield the electron self-interaction energy,

$$E_{self}^{e} = \int_0^a\int_0^b\int_0^{2\pi}\rho_e d\,\rho_e dz_e d\phi_e\ \left|\Psi_{e,\pm1/2}^{(n,n_z,m)}\right|^2\left[U_e{}^0(\mathbf{r_e}) + U_{e}{}_i^{(n)}(\mathbf{r_e})\right]. \quad (38)$$

Similarly, the hole self-interaction energy is

$$E_{self}^{h} = \int_0^a\int_0^b\int_0^{2\pi}\rho_h d\,\rho_h dz_h d\phi_h\ \left|\Psi_h^{F_z,n_z}\right|^2\left[U_h{}^0(\mathbf{r_h}) + U_{h}{}_i^{(n)}(\mathbf{r_h})\right]. \quad (39)$$

The 1D attractive potential is found by averaging terms (3) and (4) over the electron and hole probability densities,[7,11] yielding

$$V_{att}^{e,h}(|z_e - z_h|) = \int_0^a \int_0^b \int_0^{2\pi} \rho_h \rho_e d\,\rho_{e,h} dz_{e,h}\, d\phi_{e,h} \left|\Psi_{e,\pm 1/2}^{(n,n_z,m)}\right|^2 \left|\Psi_h^{F_z,n_z}\right|^2 \left[\left(U_{dir}^0(\mathbf{r_e},\mathbf{r_h}) + U_{indir}^0(\mathbf{r_e},\mathbf{r_h})\right) + \left(U_{dir}{}_i^{(n)}(\mathbf{r_e},\mathbf{r_h}) + U_{indir}{}_i^{(n)}(\mathbf{r_e},\mathbf{r_h})\right)\right] \quad (40)$$

where the first term, $\int_0^a \int_0^b \int_0^{2\pi} \rho_h \rho_e d\,\rho_{e,h} dz_{e,h}\, d\phi_{e,h} \left|\Psi_{e,\pm 1/2}^{(n,n_z,m)}\right|^2 \left|\Psi_h^{F_z,n_z}\right|^2 \left(U_{dir}^0(\mathbf{r_e},\mathbf{r_h}) + U_{indir}^0(\mathbf{r_e},\mathbf{r_h})\right)$, is the same as that given in Ref. 11, while the second term accounts for any perturbations due to image charges $q_i^{(n)}$. The 1D exciton binding (E_{bind}) energy is finally calculated using $V_{att}^{e,h}$ in a manner identical to Ref. 11. In brief, $V_{att}^{e,h}$ collapses to a 1D attractive potential within the adiabatic approximation and is subsequently used as the potential energy term in the 1D exciton Schrodinger equation. The exciton binding energy is thus determined from the eigenstate of this system, where $\left(U_{dir}{}_i^{(n)}(\mathbf{r_e},\mathbf{r_h}) + U_{indir}{}_i^{(n)}(\mathbf{r_e},\mathbf{r_h})\right)$ is a perturbation associated with image charge potentials. More information about this approach can be found in Ref. 7.

Supplementary Note 5. Weighted average of NW and *b*~160 nm NR α energies

NW and *b*~160 nm NR energies were corrected to account for their ensemble diameter distributions. A previously established sizing curve[13] was used to determine individual NW/*b*~160 nm NR radii from their corresponding single wire/rod α energies. Individual α energies were then weighted using Gaussian fits of their TEM-derived radial distributions (Supplementary Figure 2a and 2b) to obtain average α energies and standard deviations.

Supplementary Note 6. Ensemble absorption spectra of *b*~30 nm NRs and *b*≥5 μm NWs

Supplementary Figure 4 compares the ensemble absorption spectrum (open blue circles) of *b*~30 nm NRs (top) to that of corresponding NWs (bottom). It is evident that the NR ensemble absorption edge is blueshifted relative to the NW spectrum. A Gaussian fit of the NR spectrum indicates that its band edge absorption (solid green line) is ~18 meV higher than the single NR α average, which is represented by the vertical red line [E_g(single) = 1.904 eV, E_g(ensemble) = 1.886 eV]. The Gaussian peak lies within the range of observed single NR α energies. Horizontal black lines (1.904 ± 0.025 eV) indicate one standard deviation about the single NR α average. This suggests that the observed ~30 meV average blueshift of *b*~30 nm NRs is not merely a result of sampling a sub-population of smaller radii NRs.

Theoretical estimates additionally predict a ~30 meV deviation in α energies based on a 10% size distribution about *d*~6.8 nm, similar to what is observed experimentally ($\sigma_{\alpha,\text{expt}}$~25 meV). By contrast, only ~13 meV deviations are predicted based on a 30% variation in the environment dielectric constant about a mean value of ε_m=2.0. We thus conclude that the spread in *b*~30 nm single NR α energies (Figure 3 of the main text) predominantly arises from the residual size distribution of the ensemble.

Supplementary Note 7. Approximations used in the model

The following approximations/assumptions in the model potentially contribute to discrepancies between theory and observed *b*~30 nm NR average α energies:

(1) We have used a 4-band effective mass model to describe CdSe's valence band and have neglected coupling of the heavy and light hole to the split-off hole band.[13]

(2) Our model assumes that all NRs are cylindrical in shape, with aspect ratio variations due to length differences. This is motivated by the cylindrical shape of NRs seen in Supplementary Figure 2.

Supplementary Note 8. 1D-to-0D transition

Panels (a,b) in Supplementary Figure 6 demonstrate the 1D-to-0D transition of d~6.8 nm NW/NRs. The only aspect ratio-dependent contributions to their experimental transition energies are longitudinal kinetic [$E_k(b)$] and electrostatic ($|U|$) energies. Therefore the 1D-to-0D transition point is defined as the length at which these two terms balance each other out and where the dominant term switches from $|U|$ (for 1D systems, shaded grey region) to $E_k(b)$ (for 0D systems, white region). For d~6.8 nm, this occurs at b~8.5 nm (obtained from an average of $1\Sigma_{1/2}1\Sigma_e$ and $1\Sigma_{3/2}1\Sigma_e$ transition points).

Aspect ratio-dependent blueshifts are observed in NR absorption spectra due to an increase in kinetic energy from $E_k(b)$ and a decrease in the total electrostatic energy contribution. This is shown in panels (c,d) of Supplementary Figure 6 where contributions to the total kinetic energy (radial and longitudinal confinement) and total electrostatic energy (dielectric contrast and dielectric confinement) are plotted as functions of aspect ratio.

Supplementary References

---

[1] Jasieniak, J., Smith, L., van Embden, J., Califano, M. & Mulvaney, P. Re-examination of the size-dependent absorption properties of CdSe quantum dots. *J. Phys. Chem. C* **113**, 19468–19474 (2009).
[2] Yu, W. W., Qu, L., Guo, W. & Peng, X. Experimental determination of the extinction coefficient of CdTe, CdSe and CdS nanocrystals. *Chem. Mater.* **15**, 2854-2860 (2003).

[3] Kuno, M., Lee, J. K., Dabbousi, B. O., Mikulec, F. V. & Bawendi, M. G. The band edge luminescence of surface modified CdSe nanocrystallites: Probing the luminescing state. *J. Chem. Phys.* **106**, 9869-9882 (1997).
[4] Puthussery, J., Kosel,, T. H., & Kuno, M. Facile synthesis and size control of II–VI nanowires using bismuth salts. *Small* **5**, 1112–1116 (2009).
[5] Peng, Z. A. & Peng, X. Nearly monodisperse and shape-controlled CdSe nanocrystals via alternative routes: nucleation and growth. *J. Am. Chem. Soc.* **124**, 3343-3353 (2002).
[6] Efros, Al. L. & Lambrecht, W. A. Theory of light emission polarization reversal in zinc-blende and wurtzite nanowires. *Phys. Rev. B*, **89**, 035304 (2014).
[7] Shabaev, A. & Efros, Al. L. 1D exciton spectroscopy of semiconductor nanorods. *Nano Lett.* **4**, 1821–1825 (2004).
[8] Efros, Al. L. & Rosen, M. The electronic structure of semiconductor nanocrystals. *Ann. Rev. Mat. Sci.*, **30**, 475-521 (2000).
[9] Sercel, P. C., & Vahala, K. J. Analytical formalism for determining quantum-wire and quantum-dot band structure in the multiband envelope-function approximation *Phys. Rev. B*, **42**, 3690-3711 (1990).
[10] Katz, D., Wizansky, T., Millo, O., Rothenberg, E., Mokari, T. & Banin, U. Size-dependent tunneling and optical spectroscopy of CdSe quantum rods. *Phys. Rev. Lett.* **89**, 086801 (2002).
[11] Giblin, J., Vietmeyer, F., McDonald, M. P. & Kuno, M. Single nanowire extinction spectroscopy. *Nano Lett.* **11**, 3307–3311 (2011).
[12] Jackson, J. D. Classical Electrodynamics. John Wiley & Sons, Inc. New York, 1962 (p. 84).
[13] Vietmeyer, F. Chatterjee, R., McDonald, M. P. & Kuno, M. Concerted single-nanowire absorption and emission spectroscopy: Explaining the origin of the size-dependent Stokes shift in single cadmium selenide nanowires. *Phys. Rev. B* **91**, 085422 (2015).
